# Greater involvement of HIV-infected peer-mothers in provision of reproductive health services as “family planning champions” increases referrals and uptake of family planning among HIV-infected mothers

**DOI:** 10.1186/s12913-017-2386-x

**Published:** 2017-06-27

**Authors:** Peter Mudiope, Ezra Musingye, Carolyne Onyango Makumbi, Danstan Bagenda, Jaco Homsy, Mai Nakitende, Mike Mubiru, Linda Barlow Mosha, Mike Kagawa, Zikulah Namukwaya, Mary Glenn Fowler

**Affiliations:** 1 Directorate of Clinical Services - Elizabeth Glazer Pediatric Foundation, RHITES SW Project, Plot 7 Galt Road, Boma Mbarara, P.O.Box 881, Mbarara, Uganda; 2 Makerere University Walter Reed Project, Plot 42, Nakasero Road, Kampala, Uganda; 3 Makerere University-Johns Hopkins University (MU-JHU) Research Collaboration, Upper Mulago Hill Road, P.O. BOX 23491 Kampala, Uganda; 4 College of Public Health, University of Nebraska Medical Center, Omaha, USA; 5 Harvard T.H. Chan School of Public Health, Harvard University, Boston, USA; 6 Department of Obstetrics and gynecology, Makerere University College of Health Sciences, Kampala, Uganda; 7 Department of Pathology, Johns Hopkins University, 600 North Wolfe St., Carnegie, Baltimore, MD 443 USA

## Abstract

**Background:**

In 2012, Makerere University Johns - Hopkins University, and Mulago National Referral Hospital, with support from the National Institute of Health (under Grant number: NOT AI-01-023) undertook operational research at Mulago National Hospital PMTCT/PNC clinics. The study employed Peer Family Planning Champions to offer health education, counselling, and triage aimed at increasing the identification, referral and family planning (FP) uptake among HIV positive mothers attending the clinic.

**Methods:**

The Peer Champion Intervention to improve FP uptake was introduced into Mulago Hospital PMTCT/PNC clinic, Kampala Uganda. During the intervention period, peers provided additional FP counselling and education; assisted in identification and referral of HIV Positive mothers in need of FP services; and accompanied referred mothers to FP clinics. We compiled and compared the average proportions of mothers in need that were referred and took up FP in the pre-intervention (3 months), intervention (6 months), and post-intervention(3 months) periods using interrupted time series with segmented regression models with an autoregressive term of one.

**Results:**

Overall, during the intervention, the proportion of referred mothers in need of FP increased by 30.4 percentage points (*P* < 0.001), from 52.7 to 83.2 percentage points. FP uptake among mothers in need increased by over 31 percentage points (*P* < 0.001) from 47.2 to 78.5 percentage points during the intervention. There was a positive non-significant change in the weekly trend of referral β_3_ = 2.9 percentage points (*P* = 0.077) and uptake β_3_ = 1.9 percentage points (*P* = 0.176) during the intervention as compared to the pre-intervention but this was reversed during the post intervention. Over 57% (2494) mothers took up Depo-Provera injectable-FP method during the study.

**Conclusions:**

To support overstrained health care work force in post-natal clinics, peers in trained effective family planning can be a valuable addition to clinic staff in limited-resource settings. The study provides additional evidence on the utilization of peer mothers in HIV care, improves health services uptake including family planning which is a common practice in many donor supported programs. It also provides evidence that may be used to advocate for policy revisions in low-income countries to include peers as support staff especially in busy clinic settings with poor services uptake.

**Electronic supplementary material:**

The online version of this article (doi:10.1186/s12913-017-2386-x) contains supplementary material, which is available to authorized users.

## Background

Improving uptake of effective family planning (FP) and modern contraceptives and combatting HIV are components of the Sustainable Development Goals (SDG 3) [1] but remain a challenge in Sub-Saharan Africa [2]. In this region, only 28% of married (or in union) women aged 15-49 years use any effective FP method [3, 4]. The unmet need for FP (defined as women who are fecund and sexually active and report not wanting any more children or wanting to delay the next child but are not using any method of contraception) is estimated at 24% [2–4]. Family Planning is a key component of prevention of mother-to-child transmission (PMTCT) of HIV in high HIV prevalence settings [5]. Moreover unintended pregnancies irrespective of the HIV status, lead to high numbers of pregnancies, abortions, and other pregnancy and/or delivery complications; which constrain the health care systems [6]. There are also psychosocial and economic burdens to mothers and their families that come along with these unintended pregnancies [7].

Sub-Saharan Africa has the highest HIV burden with 24.7 million individuals living with HIV, accounting for 71% of all HIV-infected individuals globally, the majority of whom are women (58%) [8]. Pregnancies in HIV infected women pose additional health risks that are associated with increased mortality and morbidity for both mothers [9–13] and their infants [14–17]. Therefore, prevention of unintended pregnancies among HIV-infected women is crucial both to curb rates of mother-to-child transmission of HIV [5, 18–20] and to promote better health outcomes for HIV-infected mothers and their children [9–13] .

In Uganda, the HIV prevalence in the 15-49 year-old population is high at 7.3% and even higher at 8.3% among women in this age category [21]. At the same time, Uganda has an extremely high life time fertility rate which was 6.2 in 2011 [21] and is currently estimated at 5.8 [22]. The use of any contraceptive method among married Ugandan women aged 15-49 years is only at 30% while the unmet need for FP is 34% [4, 21] . This is of particular concern as the low use of modern FP methods has led to a high incidence of unintended pregnancies among both HIV-infected and HIV-uninfected women [23]. To increase opportunities for FP uptake, health care systems strive to provide integrated HIV, FP and other reproductive health services. However, the scale-up of FP service delivery in Uganda, has been hampered by the shortages of human resources for health (HRH) [24]: in 2010, the ratio of midwives to patients was 1: 9000, nurses to patients was 1: 1700, and doctors to patients was 1:25,000. The current staffing, skill level, and service structure within the Ugandan health care system does not provide for adequate and equitable FP services access to the population [24]. In addition, FP uptake is further hampered by patient bias, pervasive misbeliefs, and lack of information. The missed opportunity is that HIV-infected women regularly come to the health units but do not receive family planning services. These problems have been associated with limited human and financial resources; and low priority attached to FP by health care workers [24]. The rationale of employing peer family planning champion was premised on the notion that people often understand health education messages if delivered to them by a peer or someone who they perceive has or is experiencing similar situations. For this reason peer family planning champions may be more acceptable communicators than trained health workers in influencing individual behavior of peer mothers. The peer to peer interaction increases the social acceptability of health advice and services. In our previous paper we demonstrated that use of peers, influential community lay persons and Village Health Team (VHT) members increased 6-week mother-baby postnatal attendance from 37.1% at baseline to 78.5% and increased early infant diagnosis from 53.6% to 86.3% among mothers and their infants. The increase was majorly attributed to the peer mothers’ support, because the mothers reported to be more comfortable sharing their problems with the peers than the community lay persons; most mothers only disclosed their HIV status to peers and the project staff and declined to be followed by the community lay persons [25] . In another study conducted in Western Uganda, employing peer mothers resulted in increased uptake of family planning services among women living with HIV by 79% (*p* < 0.001) [26] However, in areas where PMTCT is well managed with high services uptake, peer mothers have limited additional benefit in increasing uptake of core PMTCT services. In Western Cape Province of South Africa, a study conducted in a setting with high PMTCT core services uptake, mentor mothers provided no additional increase in services uptake when compared with the standard of using traditional health worker to offer counselling. The study however, showed that the mentor mothers were more effective in conveying information and improving participants’ emotional outlook and hopefulness compared to the standard arm [27]. The ACCLIAM study conducted in Swaziland, Uganda, and Zimbabwe, to assess the effect of a package of multilevel community interventions (a social learning and action component, community dialogues, and peer-led discussion groups), on the demand for, uptake of, and retention of HIV positive pregnant/postpartum women in MCH/PMTCT service, found no incremental benefit resulting from peer-led discussion groups. This may have been associated to spurious effect of peer mother that were employed to work in the same health facilities by other projects [28]. This study therefore sought to further understand the impact of delivering FP counselling and education by mothers living with HIV (Family Planning Peer Champions) on 1) identifying women with unmet need for FP; 2) improving referrals for FP; and 3) increasing uptake of FP among HIV-infected women attending antenatal care (ANC) or postnatal care (PNC) at Mulago National Referral Hospital.

### Study site and population

The study was conducted among HIV-infected mothers attending PMTCT and PNC at Mulago National Referral Hospital. The hospital is government funded and offers a wide range of free health services including; primary, tertiary and super specialized health services. The PMTCT clinic, receives additional support from Makerere University - Johns Hopkins University Research Collaboration (MUJHU Care Ltd) and it serves families from Kampala district and surrounding areas. The PMTCT clinic, PNC clinic, the antenatal clinic (ANC) and the family planning unit are all located adjacent to each other under one roof. Such an arrangement has been described to improve access to health services across the HIV care continuum, meet users’ needs over time and create positive synergies among programs. [29, 30].

At the ANC, pregnant mothers received routine (opt out) HIV testing and counselling with same day results using rapid HIV antibody tests. The mothers newly or previously diagnosed as HIV infected were referred to the PMTCT Follow up clinic after receiving counselling and ARV prophylaxis support. These hospital clinics offer free medical services from Monday to Friday, 8.00 am to 5.00 pm. In 2012, over 33,000 pregnant women attended the ANC unit. Of these 3300 HIV infected pregnant women were identified and followed up at the PMTCT clinic. Routinely, the HIV infected women are offered basic family planning counselling and education in group sessions by midwives and PMTCT counsellors. The mothers in need of FP are encouraged and supported to take up the FP option of their choice and then referred to the adjacent Family Planning clinic. The Family Planning clinic is located in the same building with the PMTCT follow up clinic. This arrangement is intended to maximize the benefits of the FP/PMTCT integrated service delivery. However despite the availability of Family Planning Services at clinic, many mothers miss taking up Family Planning services

## Methods

This was a quasi-experimental study, evaluating a structural intervention of utilizing trained HIV-infected peer-mothers (family planning champions) to provide additional complementary FP counselling and education, assist in identification, triaging, and referral of mothers in need of FP services, and to accompany and direct referred mothers to FP clinics. At the Family planning clinic, short acting methods and surgical reversible methods were provided. The permanent surgical methods (tubal ligation and vasectomy) were provided in theatre in the adjacent block to the PMTCT/PNC clinics. The study was implemented in 2012 as a pre-post design study in three consecutive phases: pre-intervention (3 months), intervention (6 months), and post-intervention (3 months) phases. The family planning champions were recruited based on the following qualifications: previous experience working in HIV clinics, being HIV positive and having disclosed HIV status to at least one confidant, and having positive attitudes towards FP. After recruitment, the family planning champions were trained on the study protocol and their responsibilities, basic FP methods and services, HIV and FP counselling, and principles of ethical research. Two peers were recruited for each of the two FP clinics, and were supervised by two midwives on a daily basis. The four peers worked along with the clinic staff who were also oriented on the study protocol. During the intervention period, the family planning champions provided group education and additional separate counselling and actively encouraged mother in need of FP to take up the services. The family planning champions ensured compliance with general clinic attendance for follow-up care. In addition they provided education on the FP method options and emphasizing the use of dual methods to protect against further HIV risk and other sexually transmitted infections, while protecting against pregnancy as appropriate. When a mother decided to take up a method available at the PMTCT Postnatal Follow up clinic (e.g. oral contraceptives), the champion escorted the mother to the health care provider to receive the FP method. When a mother chose a method not available at the clinic, (e.g. hormonal implant or Intrauterine device), the Peer FP champions escorted the mother to the juxtaposed FP Unit and ensured the mother got the services of her choice. For those women who chose to start a new FP method, the FP clinic telephone contact was provided for use to report concerns as well as follow up at subsequent visits when possible. The family planning champions were each given $100 (UDS) as a monthly stipend for full-time work. During the pre and post intervention phases, the family planning counselling, triage and referral services were offered by midwives in the PNC/PMTCT follow-up clinic based on the standard of care. This involved offering group health education and counselling. The mothers in need of different services were then identified and referred but without active tracking or follow up to establish the outcome of the referral.

### Post-natal clinic attendance

In line with the Uganda Ministry of Health standards, HIV-infected mothers attended the PMTCT/PNC clinic at 6, 12, 24, and 36 weeks after delivery. At each of these PNC visits, mothers were offered health services including FP counselling and uptake of modern FP methods. The mothers’ use, and unmet need for FP were assessed. Mothers in need of FP (including those using FP and in need of FP refill or method change) were offered counselling on FP, referred and accompanied to FP clinics.

### Data collection and study variables

At the PMTCT follow up clinic, data on daily attendance and services uptake including; Family planning counselling and referral, were recorded by midwives and entered into a shared PMTCT data base by trained data officers. For this study, daily clinic attendance, FP counselling, need, referral and uptake, were collected using daily data collection logs by study counsellors and entered into study customized database. To exclude women who did not come through the PNC/PMTCT clinic, referred mothers were recorded in study specific logs that were used to link them at the FP clinic. At the FP clinics, only data of mothers from PNC/PMTCT clinic was collected. Data on weekly FP services utilization was summarized by the study team to inform the overall management of this implementation research. This study did not track individual mothers’ utilization of FP services. The key assessment variables in this study were: (i) proportion of FP usage among mothers attending PMTCT/PNC clinic was defined as the fraction of mothers using FP out of the total number of mothers who attended PNC/PMTCT; (ii) Proportion of mothers referred among those not using and in need FP was defined as the fraction of mothers who were referred for FP services of the total number of mothers who attended PNC/PMTCT,were not using but in need of FP; (iii) Proportion of FP uptake among mothers referred not using and in need of FP was defined as the fraction of mothers who took up FP out of the total number of mothers attended PNC/PMTCT,were not using, but in need and were referred for FP; and (iv) proportion of FP refills defined as the fraction of mothers who obtained FP refills out of the total number of mothers who attended the PNC/PMTCT and received FP whether as new acceptors or refills.

### Statistical methods

The number of women attending the FP clinics and or taking up FP during the pre-intervention, intervention and post-intervention phases, were compared using the Kruskal-Wallis rank test for equivalence of means. Comparison of the proportions for usage, referral and uptake of FP in the pre-intervention, intervention and post-intervention was done using time series based weekly aggregates as data points. The interrupted time series with segmented regression was applied to measure the intervention effect during the study [31]. There were three phases analyzed: the pre-intervention, intervention and post-intervention phases, with one change point at the start of the intervention (end of the pre-intervention) and another at the end of the intervention (start of the post-intervention). Linear models were fitted with an autoregressive term of one across the three study segments. It was therefore possible to measure separately the individual level and trend coefficients during each phase. Using the estimated models, marginal effects were used to determine the effect size of the intervention with respect to the pre- and post- intervention phases.

## Results

### Clinic attendance usage and uptake of FP at the PMTCT/PNC clinic

PMTCT/PNC clinic attendance before, during and after the intervention was stable. On a weekly basis, a mean (standard deviation- SD) of 475 (SD 129), 448 (SD = 115) and 454 (SD = 35) HIV-infected women attended the PMTCT/PNC clinic during the pre-intervention, intervention and post intervention periods respectively (Kruskal-Wallis test *p* = 0.305) [Table 1].The mean weekly uptake of FP among mothers previously not using and in need of FP was 54(SD = 16), 56(SD = 14) and 42(SD = 9) in the pre-intervention, intervention and post-intervention periods respectively (Kruskal-Wallis test *p* < 0.01). The study results showed that the majority of referred mothers took up at least one effective family planning service.

**Table 1:** Weekly average number of HIV-infected mothers not-using, referred and took up FP during the study

Indicator	Pre intervention, mean(SD)	Intervention, mean(SD)	Post-Intervention, mean(SD)
Mothers attending PMTCT/PNC	475(129)	448(115)	454(35)
Mothers not using FP	168(43)	99(42)	93(14)
Mothers not using and in need of FP	121(31)	73(30)	65(12)
Mothers not using, in need & referred for FP	63(19)	58(14)	45(9)
Mothers not using, in need, referred for and took up FP	54(16)	56(14)	42(9)

Data are presented as weekly means with standard deviations in parentheses

### Level of referral, uptake and FP refills across the study phases

The proportion of FP referrals among HIV-infected postnatal mothers attending PMTCT/PNC, not using and in need of FP was 74.7% at the start of the pre-intervention and declined at a rate of 3% (*p* = 0.005) on a week to week basis during the course of the pre-intervention period. After introducing the intervention, the proportion of FP referrals increased by 48.7 percentage points (*p* < 0.001) and it continued to increase at a rate of 2.9 percentage points (*P* = 0.077) for every week. In the post intervention period, no significant change in FP referral at onset or change in trend was observed (Table 2). The average proportion of mothers referred for FP was 52.7%, 83.2% and 72.4% in the pre intervention, intervention and post intervention respectively. On average, FP referrals increased by 30.4 percentage points (*p* < 0.001) from the pre to the intervention phase and thereafter fell by 10.8 percentage points (*p* = 0.005) in the post-intervention (Table 3).

**Table 2:** Time-series analysis for weekly FP services utilization by HIV-infected mothers during the study

	Pre-Intervention				Intervention				Post Intervention			
Indicator	Base level, β_0_	*p*-value	Base trend, β_1_	*p*-value	Change in level, β_2_	*p*-value	Change in trend, β_3_	*p*-value	Change in level, β_4_	*p*-value	Change in trend, β_5_	*p*-value
(i) Proportion (%) of FP usage among mothers attending PNC/PMTCT	64.2	<0.001	0.1	0.784	4.2	0.389	0.4	0.351	−5.4	0.01	−0.2	0.603
(ii) Proportion (%) of referrals among mothers not using and in need FP	74.7	<0.001	−3.0	0.005	48.7	<0.001	2.9	0.077	8.3	0.701	−1.5	0.375
(iii) Proportion(%) of FP uptake among mothers referred,not using and in need of FP	61.0	<0.001	−1.5	0.110	29.4	0.006	1.9	0.176	5.2	0.773	−1.9	0.338
(iv) Proportion(%) of FP refills among mothers receiving FP	45.2	<0.001	−1.7	0.002	11.9	0.020	1.5	0.010	9.2	0.015	0.1	0.860

Segmented regression of interrupted time-series models were fitted with an autoregressive term of one to measure the changes in levels and trends across the study phases. Positive coefficients represent increasing % whereas negative coefficients represent declining %. **β**_**0**_ is the proportion at the onset of the pre-intervention phase

**β**_**1**_ is the change in rate(trend) of the proportion during the pre-intervention phase. **β**_**2**_ is the change in proportion at the onset of the intervention phase. **β**_**3**_ is the change in rate(trend) of the proportion as compared to the rate (**β**_**1**_), in the pre-intervention phase. **β**_**4**_ is the change in proportion at the onset of the post-intervention phase. **β**_**5**_ is the change in rate(trend) of proportion as compared to the rate (**β**_**3**_) in the intervention phase. p- values correspond to the statistical significance level for the corresponding adjacent coefficient

**Table 3:** Average level (marginal effects) for referral, uptake and FP refills during the study phases^ℓ^

Indicator	Pre-intervention (average level)	Intervention (average level)	Post-Intervention (average level)	Effect of the Intervention vs. Pre-intervention (Intervention - Pre-intervention)	Effect of the Post Intervention vs. intervention (Post-Intervention - intervention)
(i) Proportion (%) of FP usage among mothers attending PMTCT/PNC	64.9%	77.5%	*79.8%*	12.5%*	2.3%**
(ii) Proportion of mothers referred among those not using and in need FP	52.7%	83.2%	72.4%	30.4%**	−10.8%*
(iii) Proportion of FP uptake among mothers referred not using and in need of FP	47.2%	78.5%	67.7%	31.3%**	−10.8%*
(iv) Proportion(%) of FP refills among mothers receiving FP	33.5%	33.2%	39.5%	−0.3%	6.3%*

^ℓ^Marginal effects were calculated using Segmented regression of interrupted time-series models shown in Table 2

*significant at *p* < 0.05; ** significant at *p* < 0.001;

Uptake of Family planning services: Over 61% of women in need and referred took up FP services at the onset of the pre-intervention. This proportion changed slightly by −1.5 percentage points (*p* = 0.110) per week during the pre-intervention. At the start of the intervention, the proportion of FP uptake increased by 29.4 percentage points (*p* = 0.006). In the post intervention period, no significant change in FP uptake was observed (Table 2). The average proportion of FP uptake was 47.2%, 78.5% and 67.7% in the pre-intervention, intervention and post-intervention phases respectively. On average, FP uptake in the intervention as compared to the pre-intervention phase increased by 31.3 percentage points (*p* < 0.001) but dropped by 10.8 percentage points (*P* = 0.005) thereafter in the post-intervention phase (Table 3).

### Type of FP method taken up

**Fig. 1:**
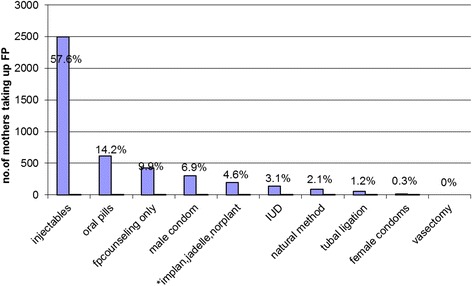
Title Family Planning service taken up by HIV infected women (*N* = 4328). “*implanon ,jadelle.norplant are aggregated together ** vasectomy and male condoms were FP methods taken up by partners of the HIV infected women”

The FP clinic provides the whole range of both long-term and short-term FP services. During the three study phases, the majority of mothers (2494; 57.6%) took up quarterly injectable Depo-Provera, followed by COCs (616, 14.2%) (Fig. 1). Together with condoms, these short-term methods accounted for 79.1% of all FP methods taken up. First-time visitors accounted for 65.5% of all mothers who took up any FP service.

## Discussion

This study showed that employing HIV-infected mothers as peer family planning champions in busy National Hospital PNC and PMTCT clinics in Kampala, Uganda improved the identification of HIV-infected mothers in need of FP services; and was also effective in improving referral, and uptake of FP services among HIV infected women. Mothers who were, not using but in need of FP that were identified and referred for FP increased by thirty percentage points during the intervention. Similarly, FP uptake among mothers in need of FP increased by thirty-one percentage points during the intervention period, but dropped by over a third during the post-intervention period. In this busy facility with low FP uptake, these results demonstrated that delivering health education and counselling through family planning champions increased the identification and referral for mothers in need of FP, compared to when the champions were not in the clinics. During the intervention period, the champions shared their own experiences, provided additional complementary counselling, offered health education, and escorted the mothers who needed family planning to the family planning provider. This process ensured that women would get essential information and not get lost between and within PMTCT/PNC and FP clinics. These results support evidence from previous studies that in busy settings with low services utilization, peer mothers’ involvement contributes to increased PMTCT services uptake [25, 26].

The 65% prevalence of FP usage observed in the pre-intervention at Mulago National Referral Hospital is over twice the 30% level of family planning uptake among married women aged 15-49 years across Uganda [4, 21]. This comparatively high level of FP usage may be explained by the fact that the study took place at a health unit where effective FP services are routinely provided. Indeed, the Mulago PMTCT/PNC clinics have been supported in staffing and training by the Makerere University - Johns Hopkins University Research Collaboration for twenty years. Also, the women in this study had recently given birth and wanted to limit or space their pregnancies as the burden of the previous pregnancy was probably more vivid and may have been a temporal motivator for FP use. During the post-intervention, the FP uptake further increased by average of 2.3 percentage points. This was because most mothers took up short term FP methods and came back for FP refills during post-intervention phase. After the intervention phase, the proportion of referrals and FP uptake dropped but the proportion of mothers returning for FP pills increased significantly by a margin of six percentage points. The mothers who received peer support in the intervention phase continued to seek and adhere to FP uptake even after the intervention stopped. The intervention actually had an immediate positive effect on FP refills as observed by the significant upward trend of FP refills during the intervention.

The success of the Peer Family Planning Champions intervention may have been due to the fact that peers had sufficient time to build rapport with mothers, thus allowing the peers to offer more individualized and specific counselling. The utilization of peers can offer an affordable and feasible task shifting solution to address frequently missed FP opportunities among HIV infected women attending PMTCT/PNC clinics. Our findings are supported by evidence from a previous study conducted at our site, that demonstrated that employing HIV-infected peers compared to community lay persons was more effective in increasing the six-week postnatal follow up of HIV-infected mothers and the early HIV testing of their infants [25]. Earlier studies and programs carried out in Uganda and Malawi, also found that use of peer mothers/educators helped in improving knowledge and retention in long-term HIV care among women living with HIV [26, 32]. Another important finding from the study was that many mothers opted for shorter-term or intermediate FP methods like hormonal pills, injectable or implants, and condoms, reflecting Uganda national statistics of the type of FP methods used, mainly because of the young women who would not otherwise take long term/permanent methods (78%) [21]. These choices raise concerns over the successful adherence to these family planning methods. Future studies using peers should also focus on how best to support mothers to consider longer term contraceptive methods.

### Study limitations and strengths

The uptake of FP by the mothers, observed in this study may not indicate increase in actual FP use especially for most short-term methods which are user dependent. However the increase in uptake of FP refills could be attributed to willingness to use and increased regular FP usage. The pre/post-intervention design used in this study is subject to temporal trends and is weaker than a randomized contemporaneous intervention design. The findings are thus based on serial cross-sectional data and do not track an individual mother’s usage over time. Also, our study did not follow mothers for more than three months in the post-intervention period, which limited our ability to assess adherence to the FP methods chosen by the women. However, strengths of the study include the large number of mothers seen in the PMTCT/PNC clinics during the 3 phases of the intervention. In addition, our study took place in a programmatic non-research setting and thus may have wide applicability to other similar settings in terms of use of peers to support family planning referrals.

## Conclusion

The task shifting use of peers in client triage, health education, linkage and referrals, at a busy National Referral Hospital in Uganda resulted in improved FP uptake during the intervention phase of the study. Based on these findings, we believe that well trained peers versed in effective family planning methods can be a valuable and cost effective addition to clinic staff in limited-resource settings with high client load and insufficient health care workforce. The study provides further support to current programs utilizing peer mothers in HIV care to improve services uptake including family planning. It also provides critical information that strongly supports the principle of meaningful involvement of people living with HIV in HIV prevention and care programs. Our results may be used to advocate for policy provisions in low-income countries to include peers as support staff, especially in busy clinic settings.

## Additional file

Additional file 1: Data set in Excel. (XLSX 18 kb)
